# Impact of Cold Ischemia Time on Allograft Survival and Policy

**DOI:** 10.1016/j.ekir.2026.106470

**Published:** 2026-03-10

**Authors:** Sai Rithin Punjala, April Logan, Ashley J. Limkemann, Navdeep Singh, Musab Alebrahim, Timothy M. Pawlik, William K. Washburn, Austin D. Schenk

**Affiliations:** 1 Division of Transplantation, Department of Surgery, The Ohio State University Wexner Medical Center, Columbus, Ohio, USA; 2 Department of Biomedical Informatics, The Ohio State University College of Medicine, Columbus, Ohio, USA; 3 Department of Surgery, The Ohio State University Wexner Medical Center, Columbus, Ohio, USA

**Keywords:** allocation out-of-sequence, cold ischemia time, expedited placement, KDPI, kidney allocation system 250 NM, kidney transplantation

## Abstract

**Introduction:**

Changes in national allocation policy and regulatory practices have led to increases in cold ischemia time (CIT) and out-of-sequence allocation (AOOS) or expedited placement (EP) of deceased donor kidney transplants in the United States. The aims of this study were to gauge the clinical relevance of the increased CIT that accompanied kidney allocation system (KAS) 250 NM (KAS250), and to measure CIT for AOOS or EP kidneys.

**Methods:**

The United Network for Organ Sharing (UNOS) data from 2007 to 2023 were analyzed across pre-KAS, KAS, and KAS250 eras. The impact of CIT on kidney graft survival and the CIT of kidneys undergoing AOOS or EP during these eras was assessed.

**Results:**

Median CIT increased from 16 hours to 19.6 hours, and the incidence of AOOS or EP increased from 0.5% to 13%. Transplants with CIT of 32 to 36 hours had a 10% increase in overall graft loss compared with CIT of 16 to 20 hours (*P* = 0.0002). For kidney donor profile index (KDPI) 20% to 34% and 35% to 85% groups, every additional hour of CIT increased the risk of graft failure by 0.5% (*P* = 0.0019) and 0.4% (*P* < 0.0001), respectively. In the KAS250 era, CIT was 4 hours longer for AOOS or EP kidneys (*P* < 0.0001). In addition, AOOS or EP kidneys with KDPI > 85% declined from 9% to 7%, whereas AOOS or EP kidneys with KDPI < 20% increased from 13% to 15% (*P* = 0.0306).

**Conclusion:**

Current allocation practices do not prioritize CIT. Extended CIT is associated with inferior long-term graft survival. AOOS or EP is designed to prevent discards but does not currently target high KDPI kidneys. Our data provide a framework to assess the relative importance of CIT in allocation policy.

## Graphical abstract

**Figure:**
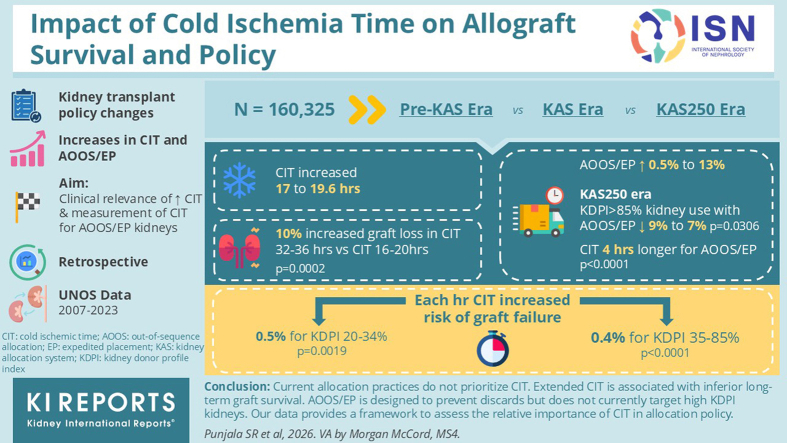

Fundamental shifts in national policy and regulatory practice are driving rapid changes in kidney transplantation in the United States. In 2021, the national kidney allocation policy radically changed with the implementation of KAS250 and the subsequent distribution of deceased donor kidneys over greater distances.^1^, ^2^, ^3^ In 2022, the Department of Health and Human Services and the Center for Medicare and Medicaid Services issued new performance metrics for organ procurement organizations (OPOs) with potential decertification for OPOs failing to identify expected numbers of organs for transplant.^4^ In 2023, the Organ Procurement and Transplantation Network’s Expeditious Taskforce explored multiple policy revisions with the bold aim of enabling 60,000 organ transplants in 2026.^5^ Concurrent with this call for a 50% increase in national transplantation volume, the Organ Procurement and Transplantation Network’s membership and professional standards committee raised the threshold for expected adverse events to discourage conservative transplantation practices.^6^ In addition, the Center for Medicare and Medicaid Services implemented the Increasing Organ Transplant Access initiative, which incentivizes increased transplantation volume and fundamentally changes the reimbursement structure for kidney transplantation.^7^

As kidney transplantation practices evolve, equity and utility are commonly at odds with efficiency. Every minute that elapses before an explanted cadaveric kidney is revascularized is deleterious to the outcome; however, efforts to distribute kidneys equitably and to maximize utility take time. Unintended consequences of KAS250 have included increased kidney discard rates, increased CITs, and increased delayed graft function.^8^^,^^9^ New OPO performance metrics have caused massive increases in organ offer volume, thereby greatly reducing allocation efficiency.^10^ Pressure to reduce kidney discard has led to dramatic increases in AOOS or “expedited” kidney placement (EP).^11^ It is too early to critically examine new membership and professional standards committee regulatory thresholds, and the impacts of Increasing Organ Transplant Access; however, competition between equity, utility, and efficiency are inevitable.

Although increasing CIT is generally perceived as unwanted inefficiency, clinically relevant timeframes are poorly defined. Our goal in this study was to determine the clinical relevance of the increase in CIT that accompanied KAS250, and similarly to consider clinically relevant CITs associated with AOOS or EP. To achieve these aims we critically examined donor variables that interact with CIT and the characteristics of kidneys undergoing AOOS or EP.

## Methods

### Data Source

Data from Standard Transplant Analysis and Research files, as maintained by the UNOS were used for retrospective cohort analysis, with follow-up through March 31, 2025, for all adult deceased donor kidney transplants performed in the United States between December 5, 2007 and January 25, 2023. Donor variables, including age, gender, ethnicity, height, weight, creatinine, history of diabetes, history of hypertension, donation after brain death or donation after circulatory death (DCD) status, hepatitis C virus (HCV) antibody status, cause of death, allocation type (local or regional or national), pumped or not pumped status, EP, and CIT; recipient variables, including age, gender, ethnicity, body mass index, calculated panel reactive antibody, primary diagnosis, previous kidney transplant status, history of diabetes, days on dialysis, functional status, human leukocyte antigen mismatch, induction agent used, and transplant year; and annualized volume for each center were obtained and included in the multivariate analysis. KDPI was included to explore interactions with CIT. These variables were used because of their availability in UNOS data within the timeframe of the study (December 5, 2007 to January 25, 2023). The reporting of calculated panel reactive antibody in UNOS started on December 5, 2007 and the formula for calculated panel reactive antibody changed on January 26, 2023. Donor HCV nucleic acid testing, cytomegalovirus and Epstein Barr virus serostatus and warm ischemic time were not included because of excessively missing data. The KDPI values were used from the September 2024 UNOS data extract, which is the last KDPI calculation based on the longstanding 2020 Kidney Donor Risk Index coefficients and the 2023 donor reference population. This is the standard KDPI formula and mapping used by UNOS until the October 2024 update, which then removed donor race and HCV serostatus from the model and would have made our values incompatible with the earlier years of our cohort. By using the September 2024 KDPI values with a clearly stated reference year (2023), we apply a single consistent KDPI scale to all recipients in our cohort, ensuring comparability across years. This approach reflects the current UNOS methodology for retrospective KDPI reporting. The data reported here have been supplied by the UNOS as the contractor for the Organ Procurement and Transplantation Network. The interpretation and reporting of these data are the responsibility of the author(s) and in no way should be seen as an official policy of or interpretation by the Organ Procurement and Transplantation Network or the United States Government.

### Study Population

Pediatric recipients, adult recipients who received living donor transplants, and multiorgan transplants were excluded. After these exclusions, annualized center volumes were calculated based on 185,391 recipients. Centers were excluded from analysis if their annualized median center volume was < 5 for the years the center was transplanting (65/269 centers, 2980/185,391 transplants, 1.6% of total transplants). Any deceased donor kidney transplant that was performed at a CIT of < 4 hours or > 36 hours was excluded, because these transplants occur infrequently and sufficient sample sizes for group analyses are not available (10,296/185,391 transplants, 5.5% of total transplants). Recipients with missing induction immunosuppression data were excluded (3852 transplants, 2% of total transplants). There were 10,296 transplants missing a variable used in multivariate analyses. Case-wise deletion for the 29 multivariate analysis variables was performed before any analysis. Pairwise deletion was used for any additional variables. There were 9649/169,974 (5.7%) missing values in the cohort of interest, including missing CIT. Multiple imputation would not substantially change the results or add meaningful value to the analysis with such a small percentage of data missing (Supplementary Table S1). Minimal bias and negligible differences (Supplementary Table S2) were discovered between variables with any missing attribute and variables with complete data.

### Study Groups and Definitions

Patients were divided into pre-KAS, KAS, and KAS250 eras based on implementation of kidney allocation policy change in the United States. All transplants between December 5, 2007 and December 3, 2014, December 4, 2014 and March 14, 2021, March 15, 2021 and December 31, 2023 were included in the pre-KAS, KAS, and KAS250 groups, respectively. AOOS or EP was identified using a definition provided by UNOS. Patients were considered to have received a kidney transplant via AOOS or EP if ≥ 1 offer before the final acceptor on the match run met the following criteria: primary refusal reason was 861 (operational - OPO), 862 or 991 (donor medical urgency), or 863 (offer not made because of EP attempt), OR primary refusal reason was 799, 898, or 998 (other specify), and the other text contains “expedited,” “open offer” or “out of sequence.” CIT values were binned into 8 groups, incrementally increasing by 4 hours (from 4 to < 8 hours to 32 to < 36 hours) and KDPI, were binned into 4 groups (< 20%, 20%–34%, 35%–85%, and > 85%).

### Statistical Methods

Medians with interquartile range for continuous variables, counts, and percentages for categorical variables were used to summarize donor and recipient variables at the transplant level, with annualized center volume summarized at the center level. Mann-Whitney U tests and Fisher exact tests were used to compare expedited transplants in the KAS era versus KAS250 era, and to compare nonexpedited versus expedited groups in the KAS250 era. Kaplan Meier estimates were fitted to graft survival, stratified by the CIT ranges. Pairwise log-rank comparisons were only performed for CIT groups of consecutive orders. Additional Kaplan Meier estimates were fitted to graft and patient survival by era, and by era and AOOS or EP status. Time-to–graft failure and time-to–patient death were censored on March 31, 2025. Time-to–graft failure was death censored.

A multivariate Cox regression model was fitted to time-to–graft failure, with an effect for CIT groupings. The noted 29 covariates were included in the model. KDPI was not included in the main model to avoid collinearity issues and maintain interpretability of the model. The variables included in the main model were ranked based on their *P*-values in the model. Forest plots were created for donor variables. Continuous variables were standardized before model fitting. The CIT with KDPI interaction was explored by fitting a multivariate cox regression model with a continuous effect for CIT and excluded all 10 covariates used to calculate KDPI. The 5-year graft survival was plotted based on medians and the most common category levels at each KDPI group (Supplementary Table S3), and reported at CIT 4, 12, 24, and 36 hours. The 5-year survival plot for the KDPI and CIT interaction displays not only the effect of KDPI but also differences in median and common categorical levels by KDPI group to capture and reflect clinically expected results. Hazard ratios (HRs) were based on increasing CIT by 1 hour, and holding all variables constant at each KDPI group, keeping all variable levels the same across the KDPI groups.

Because individual donors may contribute 2 kidneys, outcomes for recipients of kidneys from the same donor are not fully independent. In addition, within the timeframe of our study, there were 102,079 unique donors. From 58,246 (57%) donors, both kidneys were transplanted, and from 43,833 (43%) donors, only 1 kidney was transplanted. To account for this donor-level clustering without discarding data or restricting the analysis to donor pairs, we conducted a sensitivity analysis by refitting the Cox proportional hazards model using a cluster robust (sandwich) variance estimator with donors as the clustering unit. This approach preserves all available observations, allows inclusion of both donor-level and recipient-level covariates, and provides valid standard errors even when outcomes among kidneys from the same donor are correlated. Unlike donor-stratified or -paired kidney analyses, this method retains adequate statistical power and supports evaluation of donor-level covariates such as transplant year and EP (Supplementary Table S4). SAS version 9.4 (SAS Institute Inc., Cary, NC) and R version 4.4.1 (R Core Team, Vienna, Austria) software were used for all statistical analyses. This study was exempt from institutional review board approval because it was a registry analysis of publicly available deidentified data.

## Results

### Donor and Perioperative Characteristics in Pre-KAS, KAS, and KAS250 Eras

A total of 160,325 transplants were included in the analytic cohort (pre-KAS era *n* = 57,876; KAS era *n* = 73,712; KAS250 era *n* = 28,737). With the progression of time, kidney transplant volumes increased steadily between 2008 and 2022. Minor increases in the use of donors with diabetes (8% pre-KAS vs. 9% KAS250) and hypertension (28% pre-KAS vs. 30% KAS250) occurred, and major increases in DCD donor use (16% pre-KAS and 33% KAS250) and use of donors with HCV exposure (2% pre-KAS vs. 10% KAS250) occurred. Marked changes in the number of donor kidneys with a KDPI between 20% and 100% were not observed; however, the percentage of kidneys with KDPI of 0% to 19% decreased from 28% pre-KAS to 23% with KAS250. Local allocation decreased from 71% to 42% as KAS250 was implemented, and regional (14% KAS vs. 27% KAS250) and national (14% KAS vs. 31% KAS250) allocation accelerated proportionally. CIT increased from 17 hours in the KAS era to 19.6 hours with KAS250, and the incidence of out-of-sequence or EP increased markedly from 0.5% and 1.5% in the pre-KAS and KAS eras, respectively to 13% with KAS250 (Table 1). Recipient characteristics are presented in Supplementary Table S5.

**Table 1:** Summary of donor demographics across eras

Variable	Parameter value	Median (IQR) or count (%)			
		All (*N* = 160,325)	Pre-KAS (*n* = 57,876)	KAS (*n* = 73,712)	KAS250 (*n* = 28,737)
Donor demographics at the transplant level					
Donor age	yrs	40 (27–51)	41 (25–52)	39 (27–51)	40 (30–51)
Donor gender	Female	62,023 (38.7)	23,123 (40)	28,468 (39)	10,432 (36)
	Male	98,302 (61.3)	34,753 (60)	45,244 (61)	18,305 (64)
Donor ethnicity	White	109,583 (68.4)	39,966 (69)	50,310 (68)	19,307 (67)
	Black	21,766 (13.6)	7874 (14)	9903 (13)	3989 (14)
	Hispanic/Latino	23,343 (14.6)	8228 (14)	10,779 (15)	4336 (15)
	Other	5633 (3.5)	1808 (3)	2720 (4)	1105 (4)
Donor height	cm	172.0 (163.0–178.0)	170.2 (163.0–178.0)	172.0 (163.0–178.0)	172.7 (165.0–179.0)
Donor weight	kg	80.0 (67.5–95.5)	79.0 (66.0–93.7)	80.4 (67.9–96.0)	82.1 (68.9–98.1)
Donor terminal creatinine	mg/dl	0.9 (0.7–1.4)	0.9 (0.7–1.3)	0.9 (0.7–1.4)	0.9 (0.7–1.4)
Donor diabetes history	Yes	12,589 (7.9)	4446 (8)	5621 (8)	2522 (9)
Donor hypertension history	Yes	45,998 (28.7)	16,472 (28)	21,014 (29)	8512 (30)
Donor type	DBD	123,473 (77.0)	48,775 (84)	55,587 (75)	19,111 (67)
	DCD	36,852 (23.0)	9101 (16)	18,125 (25)	9626 (33)
Hepatitis C antibody	Positive	9692 (6.0)	1405 (2)	5323 (7)	2964 (10)
Cause of death	Anoxia	63,286 (39.5)	16,372 (28)	32,641 (44)	14,273 (50)
	Cerebrovascular/stroke	42,499 (26.5)	19,146 (33)	17,426 (24)	5927 (21)
	Head trauma	49,124 (30.6)	20,549 (36)	21,246 (29)	7329 (26)
	CNS tumor	635 (0.4)	283 (0)	245 (0)	107 (0)
	Other	4781 (3.0)	1526 (3)	2154 (3)	1101 (4)
Cold ischemic time	Hours	17.17 (12.08–22.39)	16.00 (11.20–21.58)	17.00 (11.96–22.18)	19.57 (15.22–23.90)
Allocation type	Local	109,587 (68.4)	44,970 (78)	52,664 (71)	11,953 (42)
	Regional	23,600 (14.7)	5163 (9)	10,552 (14)	7885 (27)
	National	27,138 (16.9)	7743 (13)	10,496 (14)	8899 (31)
Pumped	Yes	78,929 (49.2)	25,163 (43)	37,157 (50)	16,609 (58)
KDPI	0–19	42,386 (26.4)	16,020 (28)	19,650 (27)	6716 (23)
	20–34	30,818 (19.2)	10,599 (18)	14,558 (20)	5661 (20)
	35–85	80,421 (50.2)	28,816 (50)	36,658 (50)	14,947 (52)
	86+	6700 (4.2)	2441 (4)	2846 (4)	1413 (5)
Expedited placement	Yes	5070 (3.2)	303 (0.5)	1069 (1.5)	3698 (12.9)

CNS, central nervous system; DBD, donation after brain death; DCD, donation after circulatory death; IQR, interquartile range; KAS, kidney allocation system; KAS250, KAS 250 NM; KDPI, Kidney Donor Profile Index.

### CIT and Risk of Graft Failure

**Figure 1:**
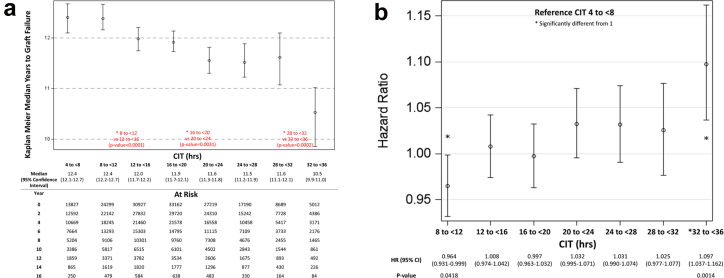
(a) Kaplan Meier 95% CIs with log-rank pairwise comparisons of CIT groups. Median kidney allograft survival gradually decreases from 12.4 years at < 12 hours CIT to 10.5 years at 32–36 hours CIT. (b) Hazard ratio of graft loss for CIT ranges with multivariate adjustment. With CIT of 4–8 hours set as reference, the hazard ratio of graft loss gradually increases with prolonged CIT. Note: Patients at risk is the same as in (a). CI, confidence interval; CIT, cold ischemia time.

**Figure 2:**
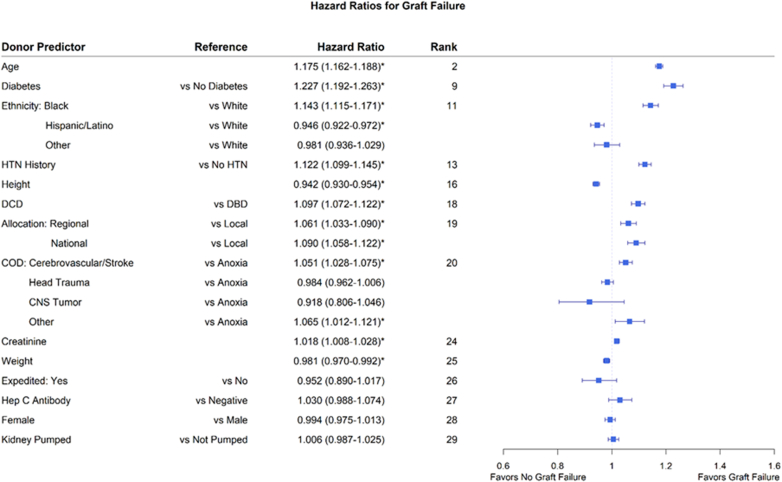
Donor predictors of graft failure from multivariate Cox regression. Forest plot depicting donor variables as predictors of graft loss. Hazard ratio >1 favors graft loss. ∗Represents statistically significant variables. CNS, central nervous system; COD, cause of death; DBD, donation after brain death; DCD, donation after circulatory death; HTN, hypertension.

An unadjusted Kaplan Meier analysis of time-to–graft failure as a function of CIT, inclusive of all donor and recipient combinations, illustrated a decrement in median graft survival from approximately 12.5 years to approximately 10.5 years as CIT increased from 4 to 36 hours (Figure 1a). On multivariate analysis after adjustment for donor and recipient variables, the risk of graft loss increased to 9.7% at CIT of 32 to 36 hours compared with CIT of 4 to 8 hours (Figure 1b). With a reference value set at CIT of 16 to 20 hours, which represented median CITs of pre-KAS, KAS and KAS250 eras, a decrease in CIT to 8 to 12 hours decreased the risk of overall graft loss by 3.3% (HR: 0.967, 95% confidence interval: 0.940–0.996, *P* = 0.0239); whereas an increase in CIT to 20 to 24, 24 to 28, and 32 to 36 hours increased the risk of overall graft loss by 3.6% (HR: 1.036, 95% confidence interval: 1.007–1.065, *P* = 0.0137), 3.5% (HR: 1.035, 95% confidence interval: 1.002–1.069, *P* = 0.0393), and 10% (HR: 1.101, 95% confidence interval: 1.047–1.158, *P* = 0.0002), respectively (Supplementary Figure S1). Donor variables strongly associated with increased risk of graft loss included older donor age, history of diabetes or hypertension in the donor, DCD donor status, Black donor ethnicity, cerebrovascular accident or stroke as cause of death, elevated terminal creatinine, and lower height and weight (Figure 2 and Supplementary Table S6). Recipient variables associated with increased risk of graft loss are presented in Supplementary Table S6.

**Figure 3:**
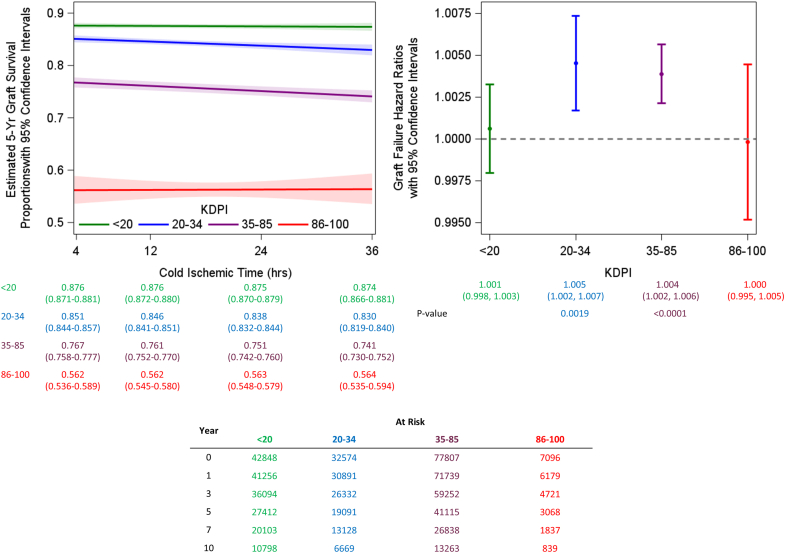
Five-year graft and patient survival probabilities and hazard ratios adjusting for the interaction of KDPI with continuous cold ischemia time. Five-year survival (left) for each KDPI is based on other covariates being set to the median or most common category level by KDPI group as shown in Supplementary Table S4, holding those covariates constant, and only allowing cold ischemia time to change. This shows results that are clinically relevant and expected for those KDPI groups. The hazard ratios (right) are based on allowing a 1-hour change in cold ischemia time while adjusting for the other covariates and holding them constant. The levels of those covariates stay consistent across all KDPI groupings to only show the effect of a 1-hour increase in cold ischemia time. KDPI, Kidney Donor Profile Index.

The relation between CIT and KDPI on 5-year graft survival probability and risk of graft loss were studied. Although, the interaction between CIT and KDPI was not significant (*P* = 0.0567). The 5-year graft survival probability decreased by 2.1% and 2.6% for KDPI 20% to 34% and 35% to 85% groups, respectively, from CIT of 4 to 36 hours. For every additional hour of CIT, the risk of overall graft failure increased by 0.5% and 0.4% for KDPI of 20% to 34% and 35% to 85% groups, respectively. CIT did not significantly affect 5-year graft survival and risk of graft loss in the KDPI < 20% group. Interestingly, CIT did not affect 5-year graft survival and risk of graft loss in the KDPI > 85% group (Figure 3).

### Characteristics of Kidneys Placed via AOOS or EP

Although only 3% of kidneys in the current study were allocated via AOOS or EP, 73% of it occurred during KAS250 era. In addition, AOOS or EP accounts for 12.9% of all kidneys allocated during KAS250. Focused comparison of expedited versus nonexpedited kidneys during KAS250 (Table 2) demonstrated that kidneys selected for AOOS or EP originated from older donors (44 vs. 40 years, *P* < 0.0001), donors with higher terminal creatinine (1.0 vs. 0.9 mg/dl, *P* < 0.0001), diabetes (11% vs. 8%, *P* < 0.0001), hypertension (37% vs. 29%, *P* < 0.0001) and DCD status (40% vs. 33%, *P* < 0.0001). Kidneys with KDPI between 35% and 85% were most likely to be placed using expedited means (63% of total) and only 7% of all kidneys placed via AOOS or EP had KDPI > 85%. Median CITs for kidneys allocated using expedited means were 4 hours longer than CITs for kidneys placed via standard allocation methods (23 hours vs. 19 hours, *P* < 0.0001) (Table 2). Focused comparison of expedited kidneys in the KAS era versus KAS250 era reveals that expedited kidneys in the KAS250 era had lower terminal creatinine (1.0 mg/dl vs. 1.2 mg/dl, *P* < 0.0001). Although the use of DCD and HCV-positive donor kidneys followed overall national trends, the use of kidneys with a KDPI > % declined from 9% in the KAS era to 7% in the KAS250 era, whereas the use of kidneys with a KDPI < 20% increased from 13% to 15% (*P* = 0.0306) (Table 2). Characteristics of recipients who received these EP kidneys are presented in Supplementary Table S7.

**Table 2:** Baseline donor characteristics comparing nonexpedited and expedited placement kidneys in the KAS250 era, and expedited placement kidneys comparing the KAS and KAS250 eras

Variable	Parameter value	KAS250 (*n* = 28,737)		*P*-value	Expedited placement kidneys (*n* = 4767)		*P*-value
		Not expedited (*n* = 25,039, 87.1%)	Expedited (*n* = 3698, 12.9%)		KAS era (*n* = 1069, 1.5%)	KAS250 era (*n* = 3698, 12.9%)	
Donor demographics at the transplant level							
Donor age	yrs	40 (30–51)	44 (33–54)	< 0.0001	46 (31–56)	44 (33–54)	0.0876
Donor gender	Female	9063 (36)	1369 (37)	0.3304	387 (36)	1369 (37)	0.6253
	Male	15,976 (64)	2329 (63)		682 (64)	2329 (63)	
Donor ethnicity	White	16,854 (67)	2453 (66)	0.0170	728 (68)	2453 (66)	0.4819
	Black	3415 (14)	574 (16)		154 (14)	574 (16)	
	Hispanic/Latino	3798 (15)	538 (15)		143 (13)	538 (15)	
	Other	972 (4)	133 (4)		44 (4)	133 (4)	
Donor height	cm	172.7 (165.0–179.1)	170.2 (163.0–178.0)	0.0007	83.5 (68.4–102.0)	83.5 (68.8–100.0)	0.9188
Donor weight	kg	82.0 (68.9–97.9)	83.5 (68.8–100.0)	0.0062	83.5 (68.4–102.0)	83.5 (68.8–100.0)	0.9188
Donor terminal creatinine	mg/dl	0.9 (0.7–1.4)	1.0 (0.7–1.8)	< 0.0001	1.2 (0.8–2.4)	1.0 (0.7–1.8)	< 0.0001
Donor diabetes history	Yes	2120 (8)	402 (11)	< 0.0001	119 (11)	402 (11)	0.8095
Donor hypertension history	Yes	7142 (29)	1370 (37)	< 0.0001	418 (39)	1370 (37)	0.2216
Donor type	DBD	16,892 (67)	2219 (60)	< 0.0001	720 (67)	2219 (60)	< 0.0001
	DCD	8147 (33)	1479 (40)		349 (33)	1479 (40)	
Hepatitis C antibody	Positive	2589 (10)	375 (10)	< 0.0001	48 (4)	375 (10)	< 0.0001
Cause of death	Anoxia	12,311 (49)	1962 (53)	< 0.0001	552 (52)	1962 (53)	< 0.0001
	Cerebrovascular/stroke	5138 (21)	789 (21)		297 (28)	789 (21)	
	Head trauma	6586 (26)	743 (20)		182 (17)	743 (20)	
	CNS tumor	93 (0)	14 (0)		2 (0)	14 (0)	
	Other	911 (4)	190 (5)		36 (3)	190 (5)	
Cold ischemic time	h	19.08 (14.80–23.28)	23.00 (18.75–27.28)	< 0.0001	23.15 (18.40–28.00)	23.00 (18.75–27.28)	0.6190
Allocation type	Local	10,805 (43)	1148 (31)	< 0.0001	301 (28)	1148 (31)	0.1103
	Regional	6972 (28)	913 (25)		259 (24)	913 (25)	
	National	7262 (29)	1637 (44)		509 (48)	1637 (44)	
Pumped	Y	14,109 (56)	2500 (68)	< 0.0001	637 (60)	2500 (68)	< 0.0001
KDPI, %	0–19	6173 (25)	543 (15)	< 0.0001	135 (13)	543 (15)	0.0306
	20–34	5080 (20)	581 (16)		168 (16)	581 (16)	
	35–85	12,618 (50)	2329 (63)		670 (63)	2329 (63)	
	86+	1168 (5)	245 (7)		96 (9)	245 (7)	

CNS, central nervous system; DBD, donation after brain death; DCD, donation after circulatory death; KAS, kidney allocation system; KAS250, KAS 250 NM; KDPI, Kidney Donor Profile Index.

### Graft Survival by Era and With/Without EP

**Figure 4:**
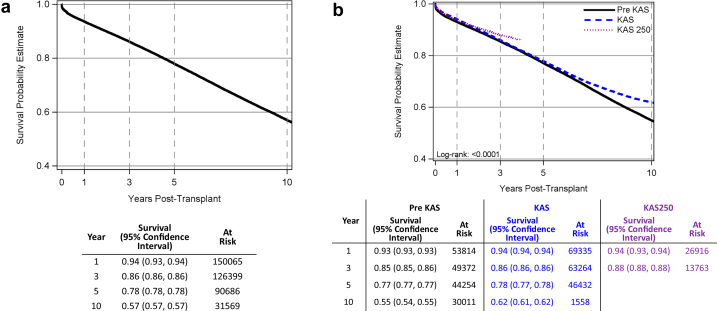

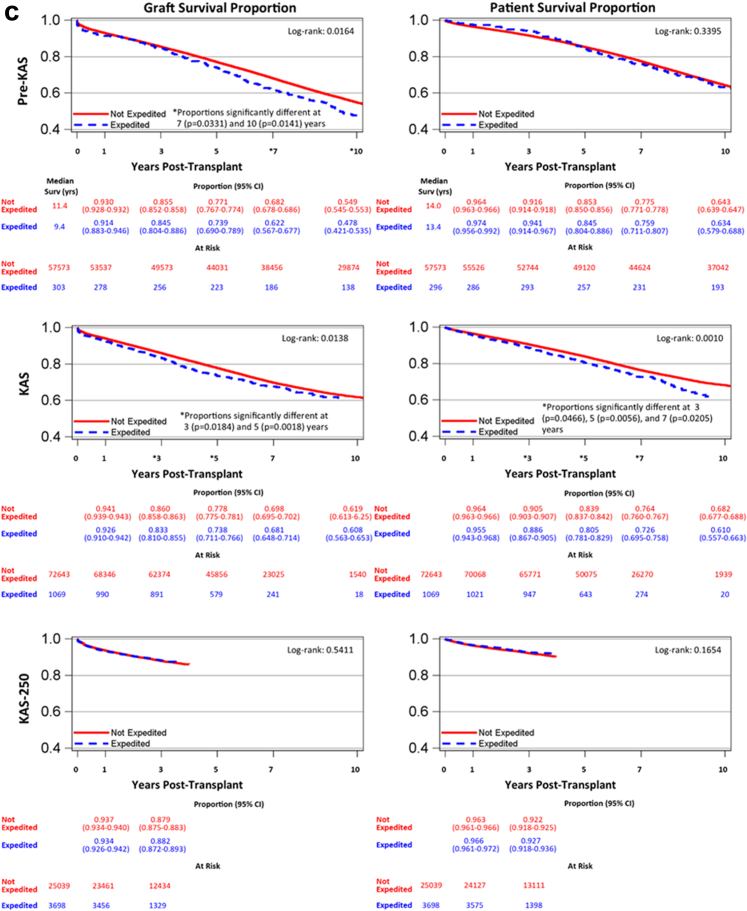
(a) Graft survival probability in the study period. Kaplan-Meier curve and 95% CIs of 1-, 3-, 5- and 10-year graft survival during the study period. (b) Graft survival probability by era. Kaplan-Meier curves and 95% CIs of graft survival compared by pre-KAS, KAS, and KAS250 eras. Graft survival improved with progression of eras. (c) Graft and patient survival by expedited placement. Kaplan-Meier curves and 95% CIs of graft and patient survival by expedited placement and pre-KAS, KAS, and KAS250 eras. Values were censored at United Network for Organ Sharing follow-up date of March 31, 2025. If patients died, graft survival was censored at patient death. Kaplan-Meier median graft survival is only available for pre-KAS, because 50% of the cohort is needed to have a graft failure. CI, confidence interval; KAS, kidney allocation system; KAS250, KAS 250 NM.

The overall estimated graft survival at 1, 3, 5, and 10 years was 94%, 86%, 78%, and 57%, respectively (Figure 4a). When stratified by pre-KAS, KAS, and KAS250 eras, long-term graft survival improved with time through the eras (log-rank *P* < 0.0001) (Figure 4b). Comparisons of graft survival by placement type (AOOS or EP vs. nonexpedited) in the KAS250 era were not associated with a difference at 1 and 3 years (log-rank *P* = 0.5411) (Figure 4c).

## Discussion

The current study reports a comprehensive analysis of the relationship between CIT and graft survival incorporating data that span 3 sequential policies for kidney allocation in the United States. As expected, there were increases in kidney volume over time, including increased use of kidneys from DCD donors and donors with HCV exposure. Of note, change from KAS to KAS250 was accompanied by an approximately 2.5 hour increase in CIT. Use of AOOS or EP dramatically increased in the KAS250 era, which represented a 7.6-fold increase compared with the KAS era.

The data reinforce the well-worn and well-accepted adage that “every minute counts” in transplantation and add clinical context to this observation. The median time-to–graft failure decreased from 12.5 to 10.5 years as CIT increased from 4 to 36 hours. Given that 1 in 10 renal transplants performed in the KAS250 era was a retransplant, extending the median survival of each organ transplanted by 1 to 2 years is not trivial. If national organ allocation policy were crafted to maximize graft survival by minimizing CIT, it would be necessary to reduce CIT to ≤12 hours to observe a meaningful effect. Median CITs ≤ 12 hours are currently not feasible in the current paradigm in which kidney allocation occurs postdonation and typically after biopsy and pump parameters become available. From the practical standpoint of organ acceptance, reduced graft longevity associated with prolonged CIT must be balanced against the high mortality associated with dialysis; and in this light, the decision to proceed with transplant is typically favorable. Furthermore, it is important to consider which priorities in allocation are taking precedence over CIT. Presumably, these are the factors related to equity in allocation ensuring that patients in all locations and with varied levels of sensitization have equal access to transplant.

In the current study, we investigated whether the association between CIT and graft longevity is fixed or varies with the type of renal allograft under consideration. Interestingly, there were differences in the relationship between CIT and graft survival when KDPI was used to stratify donors. Prolonged CIT had no impact on graft survival for high quality organs (KDPI < 20%). Kidney grafts with KDPI of 20% to 34% and 35% to 85% had worse graft survival outcomes with prolonged CIT, where every additional hour of CIT increased the risk of graft loss. Paradoxically, kidneys with KDPI > 85% were not associated with reduced graft survival with prolonged CIT. A possible explanation for this finding could be the potential for selection bias in which only the best organs among kidneys with high-risk features were handpicked for transplant. This underlying selection bias may plausibly contribute to the observed nonsignificant interaction between KDPI and CIT. Cashion *et al.*^12^ have explored the interaction of KDPI and CIT on outcomes after kidney transplantation. Their findings suggest that worse short-term outcomes are observed with prolonged CIT on low (< 20%) and intermediate (21%–85%) KDPI kidneys. In agreement with our findings, prolonged CIT did not have an impact on short- and long-term outcomes among high KDPI (> 85%) kidneys. In addition, it is possible that additional unfavorable characteristics associated with higher KDPI allografts override the impact of CIT.

The number of kidneys allocated via AOOS or EP has grown exponentially during the KAS250 era. This increase is partially attributable to the sharp increase in organ offers generated by KAS250^10^ and greatly amplified by new pressures applied to OPOs by the Center for Medicare and Medicaid Services.^4^ Although AOOS or EP does theoretically offer the possibility to reduce CIT, kidneys placed via expedited means in this study had a median CIT 4 hours greater than those allocated via standard means. Currently, AOOS or EP is not serving to “optimize” CIT on “at-risk” kidneys.

Curiously, only 6% of all kidneys allocated via EP had KDPI > 85%. Kidney nonuse is observed more often among high KDPI (> 85%) kidneys, which has been on the rise over the last few years.^13^, ^14^, ^15^, ^16^ It is possible that scrutiny on organ donation rates may have led to increase in workup and donations from older “high risk” donors, changing the donor pool, thereby contributing to the increase in kidney nonuse.^15^^,^^16^ The survival advantage–associated high KDPI kidneys, as compared with remaining on dialysis should not be overlooked.^15^^,^^17^ Simulation models have demonstrated that nonuse rate of high-risk kidneys would be reduced if expedited early or if offered to multiple centers at once, nationally.^18^^,^^19^ In the current study, there was no difference in survival between standard allocation kidneys and AOOS or EP kidneys in KAS250 era. This finding was not surprising because AOOS or EP was not being applied to the most “vulnerable” high KDPI kidneys.

A key limitation of this study is the absence of anatomic, biopsy, and pump data. These variables likely influence OPO decisions regarding the use of AOOS or EP but were beyond the study’s scope. The reasons for kidney nonuse are multifactorial and include donor quality, donor history of diabetes, biopsy findings, surgical damage to the kidneys, change in donor pool, thresholds on the quality of the kidneys used by transplant centers; all of which impact the threshold for risk among transplant physicians.^16^^,^^20^, ^21^, ^22^ Another limitation was the absence of data on kidney discards, machine perfusion duration, use of normothermic regional perfusion, and donor care unit practices. EP policies have generally been crafted to minimize discard of “at risk” kidneys rather than to maximize graft longevity by optimizing variables such as CIT. Ethical implications of EP such as outcomes in patients on the kidney waitlist who were bypassed, and the potential to introduce geographic disparities as an indirect consequence of kidneys allocated OOS by select OPOs to select transplant centers has not been explored in this study.^11^^,^^23^^,^^24^ Another limitation is that we do not have the actual KDPI or Kidney Donor Risk Index values from the year of transplant. Rather, we have retro-calculated KDPI and Kidney Donor Risk Index using the 2023 reference population and 2020 coefficients, before the 2024 Kidney Donor Risk Index recalibration. We expect these to behave similarly to historical values.

In addition, unmeasured allocation factors may confound the observed association between CIT and graft survival. A donor-stratified Cox analysis was considered; however, paired kidney comparisons are most informative only for moderate CIT differences and lose statistical power for larger differences. In our dataset, the majority of kidneys from the same donor differed in CIT by approximately 5 hours (median: 4.8 hours; interquartile range: 2.7–7.8 hours), limiting the ability of a paired design to evaluate the full CIT range. This approach would require excluding 43,833 donors (43% of the cohort) who provided a single kidney for transplantation, further reducing statistical power. In addition, a paired design does not allow evaluation of donor-level covariates such as EP strategies or transplant year because these variables do not vary within donor pairs. A frailty model was considered but was not computationally feasible because of the large number of donors and model covariates. As an alternative, we performed a sensitivity analysis using a cluster robust (sandwich) variance estimator to account for donor-level clustering. This approach preserved all available observations, allowed examination across the full CIT distribution, supported inclusion of donor-level variables, and produced results consistent with the primary multivariable Cox model.

In summary, this study provides quantitative data on CIT thresholds that can inform discussions of allocation trade-offs. It cannot be definitively determined whether the CIT increase is attributable to the KAS250 policy, the Center for Medicare and Medicaid Services regulatory pressure, or changes in the underlying donor pool. When CIT is considered in the context of larger policy revisions and current standards, we suggest that only CIT shifts of 8 or 12 hours would significantly impact clinical outcomes. We do acknowledge, however, that CIT is just an important variable in the incredibly complex discussion of allocation. Although EP was developed to decrease organ discard rather than specifically optimize CIT, our findings show that EP has not reduced CIT nor has it increased the use of the most “at risk” organs. Substantial revisions of the EP policy would be required to shift the purpose of EP to optimizing graft longevity along with preventing discards. In liver transplantation, normothermic oxygenated machine perfusion effectively “stops time,” and time-to-pump has replaced CIT as the relevant measure of time in liver transplantation. Similar technologies will radically alter the landscape in kidney transplantation. Until then, CIT remains a critical variable in the ongoing struggle between equity, utility, and efficiency.

## Disclosure

All the authors declared no competing interests.

## Supplementary Material

Supplementary File (PDF) **Figure S1.** Hazard ratio for CIT ranges with multivariate adjustment with reference value set at CIT of 16 to 20 hours (observed median CITs across eras). **Table S1.** Frequency and distribution of missing data. **Table S2.** Missingness bias table for subsets of data falling within cold ischemic range of 4 to 36 hours. **Table S3.** Multivariate Cox regression KDPI levels used for 5-year survival with KDPI and CIT interaction. **Table S4.** Multivariate models with cluster-robust standard errors by donor. **Table S5.** Summary of recipient demographics along with center volume across eras. **Table S6.** Multivariate Cox regression results based on standardized data. **Table S7.** Baseline recipient characteristics comparing nonexpedited and expedited placement kidneys in the KAS250 era, and expedited placement kidneys comparing the KAS and KAS250 eras.

## Footnotes

Supplementary File (PDF)
**Figure S1.** Hazard ratio for CIT ranges with multivariate adjustment with reference value set at CIT of 16 to 20 hours (observed median CITs across eras). **Table S1.** Frequency and distribution of missing data. **Table S2.** Missingness bias table for subsets of data falling within cold ischemic range of 4 to 36 hours. **Table S3.** Multivariate Cox regression KDPI levels used for 5-year survival with KDPI and CIT interaction. **Table S4.** Multivariate models with cluster-robust standard errors by donor. **Table S5.** Summary of recipient demographics along with center volume across eras. **Table S6.** Multivariate Cox regression results based on standardized data. **Table S7.** Baseline recipient characteristics comparing nonexpedited and expedited placement kidneys in the KAS250 era, and expedited placement kidneys comparing the KAS and KAS250 eras.
