# Supplementary material for: Impact of Cold Ischemia Time on Allograft Survival and Policy
Source: Kidney Int Rep. 2026 Mar 10;11(5):106470. doi: 10.1016/j.ekir.2026.106470 (PMC13088964; doi:10.1016/j.ekir.2026.106470)
Supplement: Supplementary File (PDF) — Figure S1. Hazard ratio for CIT ranges with multivariate adjustment with reference value set at CIT of 16 to 20 hours (observed median CITs across eras). Table S1. Frequency and distribution of missing data. Table S2. Missingness bias table for subsets of data falling within cold ischemic range of 4 to 36 hours. Table S3. Multivariate Cox regression KDPI levels used for 5-year survival with KDPI and CIT interaction. Table S4. Multivariate models with cluster-robust standard errors by donor. Table S5. Summary of recipient demographics along with center volume across eras. Table S6. Multivariate Cox regression results based on standardized data. Table S7. Baseline recipient characteristics comparing nonexpedited and expedited placement kidneys in the KAS250 era, and expedited placement kidneys comparing the KAS and KAS250 eras. [file mmc1.pdf]

Supplementary Material

Supplement to: Sai Rithin Punjala, April J Logan, Ashley J Limkemann, et al., Cold Ischemia Time Impact on Kidney Allograft Survival and Policy Implications

This supplement has been provided by the authors to give readers additional information about the work.

Table of contents

Figure S1 Hazard Ratio for CIT ranges with Multivariate Adjustment with reference value set at CIT of 16-20 hours (observed median CITs across eras) .... **page 3**

Table S1 Frequency and Distribution of Missing Data .... **page 4**

Table S2 Missingness bias table for subsets of data falling within cold ischemic range of 4 to 36 hours .... **page 5**

Table S3 Multivariate Cox Regression KDPI Levels Used for 5-Year Survival with KDPI and CIT Interaction.. **page 7**

Table S4 Multivariate Models with Cluster-Robust Standard Errors by Donor .... **page 8**

Table S5 Summary of Recipient Demographics along with Center Volume Across Eras .... **page 10**

Table S6 Multivariate Cox Regression Results Based on Standardized Data .... **page 12**

Table S7 Baseline recipient characteristics comparing non-expedited and expedited placement kidneys in the KAS250 era, and expedited placement kidneys comparing the KAS and KAS 250 eras .... **page 14**

Figure S1 Hazard Ratio for CIT ranges with Multivariate Adjustment with reference value set at CIT of 16-20 hours (observed median CITs across eras)

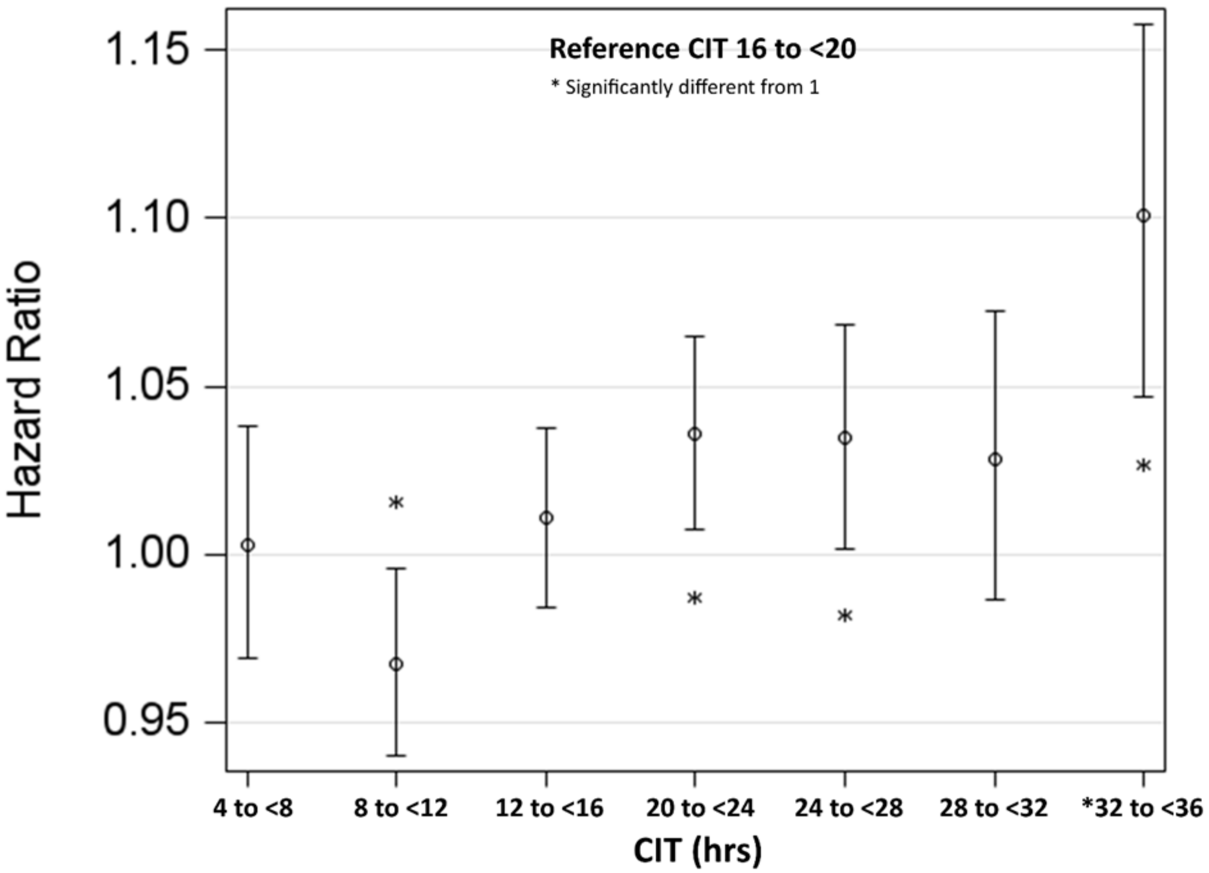

	4 to <8	8 to <12	12 to <16	20 to <24	24 to <28	28 to <32	*32 to <36
**HR (95% CI)**	1.003 (0.969-1.038)	0.967 (0.940-0.996)	1.011 (0.984-1.038)	1.036 (1.007-1.065)	1.035 (1.002-1.069)	1.029 (0.987-1.072)	1.101 (1.047-1.158)
**P-value**		0.0239		0.0137	0.0393		0.0002

Table S1. Frequency and Distribution of Missing Data (N=168,646, excluding missing CIT)

**Donor Variables**	**# Missing (%)**	**Recipient Variables**	**# Missing (%)**
Donor age	0 (0.0%)	Expedited Placement	31 (0.0%)
Donor gender	0 (0.0%)	Age	0 (0.0%)
Donor ethnicity	0 (0.0%)	Gender	0 (0.0%)
Donor height	9 (0.0%)	Ethnicity	0 (0.0%)
Donor weight	175 (0.1%)	BMI	72 (0.0%)
Donor terminal creatinine	10 (0.0%)	cPRA level	0 (0.0%)
Donor diabetes history	1450 (0.9%)	Primary Diagnosis	12 (0.0%)
Donor hypertension history	1628 (1.0%)	Previous Kidney Tx	0 (0.0%)
Donor type	0 (0.0%)	Diabetes history	405 (0.2%)
Donor Hep C Antibody	22 (0.0%)	Dialysis	319 (0.2%)
Donor cause of death	0 (0.0%)	Days on Dialysis	1418 (0.8%)
Cold Ischemic Time	0 (0.0%)	Functional Status	4456 (2.6%)
Allocation Type	0 (0.0%)	HLA mismatch	0 (0.0%)
Pumped	1 (0.0%)	Induction Group	0 (0.0%)
KDPI	355 (0.2%)	Transplant Year	0 (0.0%)
Overall Missing		8321 (4.9%)	

Abbreviations: Hep C, hepatitis C virus; KDPI, Kidney Donor Profile Index; BMI, body mass index; cPRA, calculated panel reactive antibody; HLA, human leukocyte antigen

Note: Of 185,391 recipients, 1,328 (0.7%) had a missing cold ischemic time, 9,060 (4.9%) had cold ischemic time falling outside of the 4-to-36-hour range, 3,852 (2.1%) were on an induction immunosuppression not considered to be depleting or non-depleting, and 2,980 (1.6%) were from centers with volumes of less than 5 annualized median transplants. Counting missing cold ischemic time, there were 9,649/169,974 (5.7%) missing values.
We want our study to reflect CIT ranging from 4 to 36 hours. If CIT is missing, we don’t know if differences we see are based on kidneys falling OUTSIDE of the CIT range or because of some reason connected to the result being missing.

Table S2. Missingness bias table for subsets of data falling within cold ischemic range of 4 to 36 hours.

Variable	Category	Not Missing N=160,325 Mean (SD)	Missing N	Missing Mean (SD)	Common Language Effect Size
**Donor**					
Donor age		39 (16)	8321	40 (15)	0.07
Donor gender		0.61 (0.49)	8321	0.64 (0.48)	0.04
Donor ethnicity	White	0.68 (0.47)	8321	0.66 (0.47)	0.04
	Black	0.14 (0.34)	8321	0.15 (0.36)	0.04
	Hispanic/Latino	0.15 (0.35)	8321	0.14 (0.35)	0.00
	Other	0.04 (0.18)	8321	0.04 (0.20)	0.03
Donor height		168.9 (17.9)	8312	169.7 (16.0)	0.03
Donor weight		81.7 (25.3)	8146	81.4 (23.7)	0.01
Donor terminal creatinine		1.2 (1.1)	8311	1.3 (1.1)	0.01
Donor diabetes history		0.08 (0.27)	6871	0.08 (0.27)	0.00
Donor hypertension history		0.29 (0.45)	6693	0.31 (0.46)	0.04
Donor type		0.23 (0.42)	8321	0.19 (0.40)	0.06
Hep C antibody		0.06 (0.24)	8299	0.06 (0.25)	0.01
Donor cause of death	Anoxia	0.39 (0.49)	8321	0.38 (0.48)	0.03
	Cerebrovascular/Stroke	0.27 (0.44)	8321	0.28 (0.45)	0.03
	Head Trauma	0.31 (0.46)	8321	0.31 (0.46)	0.01
	CNS Tumor	0.00 (0.06)	8321	0.00 (0.05)	0.01
	Other	0.03 (0.17)	8321	0.03 (0.16)	0.01
Cold Ischemic Time		17.54 (7.06)	8321	17.42 (7.09)	0.01
Allocation Type	Local	0.68 (0.47)	8321	0.69 (0.46)	0.00
	Regional	0.15 (0.35)	8321	0.13 (0.34)	0.03
	National	0.17 (0.37)	8321	0.18 (0.38)	0.02
Pumped		0.49 (0.50)	8320	0.47 (0.50)	0.03
KDPI		39.70 (24.81)	7966	42.77 (25.13)	0.09
**Recipient**					
Expedited Placement		0.03 (0.17)	8290	0.03 (0.17)	0.01
Age		53 (13)	8321	53 (13)	0.01
Gender		0.60 (0.49)	8321	0.59 (0.49)	0.01
Ethnicity	White	0.39 (0.49)	8321	0.38 (0.49)	0.02
	Black	0.34 (0.47)	8321	0.36 (0.48)	0.03
	Hispanic/Latino	0.18 (0.38)	8321	0.17 (0.37)	0.02
	Other	0.09 (0.29)	8321	0.10 (0.30)	0.01
BMI		28.41 (5.43)	8249	28.30 (5.43)	0.01
cPRA level		24 (36)	8321	25 (37)	0.03
Primary Diagnosis	Type I diabetes	0.03 (0.16)	8309	0.03 (0.16)	0.01
	Type II diabetes	0.26 (0.44)	8309	0.24 (0.43)	0.02
	HTN	0.24 (0.43)	8309	0.23 (0.42)	0.01
	PKD	0.07 (0.26)	8309	0.07 (0.26)	0.00
	Graft failure	0.07 (0.25)	8309	0.07 (0.26)	0.02
	IgA nephropathy	0.04 (0.20)	8309	0.04 (0.19)	0.01
	SLE	0.03 (0.17)	8309	0.03 (0.17)	0.00
	Other	0.27 (0.44)	8309	0.28 (0.45)	0.02
Previous Kidney Tx		0.12 (0.32)	8321	0.13 (0.34)	0.03

Diabetes history		0.37 (0.48)	7916	0.37 (0.48)	0.00
Dialysis		0.90 (0.30)	8002	0.91 (0.29)	0.02
Time on Dialysis		1516 (1262)	6903	1582 (1283)	0.04
Functional Status	10 to 40%	0.02 (0.15)	3865	0.03 (0.16)	0.01
	50 to 70%	0.41 (0.49)	3865	0.38 (0.48)	0.05
	80 to 100%	0.57 (0.50)	3865	0.60 (0.49)	0.04
HLA mismatch	0	0.06 (0.24)	8321	0.07 (0.25)	0.01
	1 to 4	0.47 (0.50)	8321	0.47 (0.50)	0.00
	5 to 6	0.47 (0.50)	8321	0.46 (0.50)	0.00
Induction Group	Depleting	0.72 (0.45)	8321	0.70 (0.46)	0.03
	Nondepleting	0.15 (0.36)	8321	0.14 (0.35)	0.03
	Both	0.03 (0.16)	8321	0.03 (0.17)	0.01
	None	0.10 (0.30)	8321	0.13 (0.34)	0.06
Transplant Year		2016.06 (4.35)	8321	2015.27 (4.84)	0.12

Abbreviations: Hep C, hepatitis C virus; KDPI, Kidney Donor Profile Index; BMI, body mass index; cPRA, calculated panel reactive antibody; HLA, human leukocyte antigen

Note: We exclude missing cold ischemic time from this analysis because we would not be able to determine if differences that we see is due to the cold ischemic time being out of range or missing.

Table S3 Multivariate Cox Regression KDPI Levels Used for 5-Year Survival with KDPI and CIT Interaction

Variable	KDPI			
Donor	<20	20-34	35-85	86-100
Age				
Height (cm)				
Weight (kg)				
Creatinine				
Ethnicity				
History of Hypertension				
Diabetes				
Donor Hepatitis C Virus Antibody				
Cause of Death				
Non-Heart Beating				
HLA Mismatch	1 to 4	1 to 4	5 to 6	5 to 6
Gender	Male	Male	Male	Female
KDPI				
Kidney Pumped	No	No	Yes	Yes
Recipient				
Age	47	53	58	64
Body Mass Index	27.9	28.2	28.1	27.7
End cPRA	0	0	0	0
Gender	Male	Male	Male	Male
Ethnicity	White	White	White	White
Diabetes	No	No	No	Yes
Diagnosis	Other	Other	Type II Diabetes	Type II Diabetes
Days on Dialysis	1240	1413	1381	1174
Previous Kidney Transplant	No	No	No	No
Cold Ischemia Time				
Induction	Depleting	Depleting	Depleting	Depleting
Expedited Placement	No	No	No	No
Allocation	Local	Local	Local	Local
Functional Status	80 to 100%	80 to 100%	80 to 100%	80 to 100%
Transplant Year	2016	2017	2017	2017
Annualized Center Volume	85	89	95	105

Abbreviations: KDPI, Kidney Donor Profile Index; cPRA, calculated panel reactive antibody; HLA, human leukocyte antigen

Table S4. Multivariate Models with Cluster-Robust Standard Errors by Donor.

Model 1: Multivariate with Categorical CIT and KDPI components			
**Variable**	**HR and 95% CI**	**p-value**	**Rank**
Recipient Age, years	1.228 (1.214,1.242)	<0.0001	1
Recipient Ethnicity (ref=White)		<0.0001	2
Black	0.994 (0.974,1.016)	0.6020	
Hispanic/Latino	0.743 (0.722,0.763)	<0.0001	
Other	0.712 (0.688,0.737)	<0.0001	
Donor Age, years	1.175 (1.162,1.189)	<0.0001	3
Days on Dialysis	1.120 (1.110,1.131)	<0.0001	4
Diagnosis at Time of Listing (ref= Type II diabetes)		<0.0001	5
Type I diabetes	1.029 (0.981,1.080)	0.2380	
Hypertension	0.943 (0.913,0.974)	0.0003	
Polycystic Kidney Disease	0.644 (0.615,0.674)	<0.0001	
Graft failure	0.879 (0.832,0.928)	<0.0001	
IgA nephropathy	0.654 (0.614,0.696)	<0.0001	
Systemic Lupus Erythematous	1.038 (0.974,1.106)	0.2504	
Other	0.913 (0.884,0.944)	<0.0001	
Transplant Year	0.891 (0.881,0.901)	<0.0001	6
Recipient Diabetes (ref=No)	1.308 (1.271,1.346)	<0.0001	7
Functional Status (ref=80% to 100%)		<0.0001	8
10% to 40%	1.272 (1.204,1.344)	<0.0001	
50% to 70%	1.126 (1.106,1.146)	<0.0001	
Donor Diabetes (ref=No)	1.227 (1.190,1.265)	<0.0001	9
Recipient Gender (ref=Female)	0.886 (0.870,0.903)	<0.0001	10
Recipient Body Mass Index, kg/m^2	1.055 (1.046,1.065)	<0.0001	11
Donor Ethnicity (ref=White)		<0.0001	12
Black	1.143 (1.114,1.172)	<0.0001	
Hispanic/Latino	0.946 (0.921,0.972)	<0.0001	
Other	0.981 (0.935,1.029)	0.4380	
Donor Hypertension History (ref=No)	1.122 (1.099,1.146)	<0.0001	13
Human leukocyte antigen mismatch (ref=0)		<0.0001	14
1 to 4	1.147 (1.104,1.192)	<0.0001	
5 to 6	1.221 (1.173,1.270)	<0.0001	
Induction Group (ref=Depleting)		<0.0001	15
Non-Depleting	1.032 (1.008,1.056)	0.0084	
Depleting & Non-Depleting	1.129 (1.075,1.185)	<0.0001	
None	1.144 (1.114,1.174)	<0.0001	
Donor Height, cm	0.942 (0.930,0.954)	<0.0001	16
Calculated panel reactive antibody, %	1.048 (1.037,1.060)	<0.0001	17
Non-Heart Beating Donor (ref=No)	1.097 (1.072,1.123)	<0.0001	18
Allocation Type (ref=Local)		<0.0001	19
Regional	1.061 (1.032,1.091)	<0.0001	
National	1.090 (1.058,1.123)	<0.0001	
Donor cause of death (ref=Anoxia)		<0.0001	20
Cerebrovascular /Stroke	1.051 (1.027,1.076)	<0.0001	
Head Trauma	0.984 (0.962,1.006)	0.1473	
CNS Tumor	0.918 (0.801,1.052)	0.2164	
Other	1.065 (1.010,1.123)	0.0191	

Previous Kidney Transplant (ref=No)	1.102 (1.059,1.146)	<0.0001	21
Cold Ischemia Time Range (ref=4 to <8), hours		<0.0001	22
8 to <12	0.964 (0.932,0.998)	0.0394	
12 to <16	1.008 (0.974,1.042)	0.6588	
16 to <20	0.997 (0.963,1.032)	0.8636	
20 to <24	1.032 (0.996,1.071)	0.0852	
24 to <28	1.031 (0.990,1.074)	0.1346	
28 to <32	1.026 (0.976,1.077)	0.3147	
32 to <36	1.098 (1.036,1.162)	0.0015	
Annualized Center Volume, n	0.979 (0.969,0.989)	<0.0001	23
Donor Creatinine, mg/dl	1.018 (1.008,1.028)	0.0002	24
Donor Weight, kg	0.981 (0.970,0.992)	0.0010	25
Expedited Placement (ref=No)	0.952 (0.889,1.019)	0.1575	26
Donor Hepatitis C Virus Antibody (ref=Negative)	1.030 (0.988,1.074)	0.1672	27
Donor Gender (ref=Male)	0.994 (0.975,1.014)	0.5534	28
Kidney Pumped (ref=No)	1.006 (0.986,1.026)	0.5623	29
**Model 2: Continuous CIT and Categorical KDPI**			
Hazard Ratio for one-hour change in CIT by KDPI level		0.0606	
KDPI<20	1.001 (0.998-1.003)	0.6501	
20-24	1.005 (1.002-1.007)	0.0019	
35-85	1.004 (1.002-1.006)	<0.0001	
86-100	1 (0.995-1.005)	0.9400	

Abbreviations: CIT,cold ischemia time; KDPI, Kidney Donor Profile Inde

Table S5. Summary of Recipient Demographics along with Center Volume Across Eras

Variable	Parameter Value	Median (IQR) Or Count (%)			
		All (n=160,325)	Pre KAS (n=57,876)	KAS (n=73,712)	KAS 250 (n=28,737)
**Recipient Demographics**					
Recipient Age	Years	55 (44-64)	55 (45-63)	55 (44-64)	55 (44-64)
Gender	Female	63726 (39.7)	22827 (39)	29412 (40)	11487 (40)
	Male	96599 (60.3)	35049 (61)	44300 (60)	17250 (60)
Ethnicity	White	62711 (39.1)	25530 (44)	27409 (37)	9772 (34)
	Black	54129 (33.8)	18703 (32)	25342 (34)	10084 (35)
	Hispanic/Latinx	28769 (17.9)	8916 (15)	13908 (19)	5945 (21)
	Other	14716 (9.2)	4727 (8)	7053 (10)	2936 (10)
Body mass index	kg/m^2	28.0 (24.3-32.2)	27.9 (24.3-32.0)	28.1 (24.3-32.2)	28.2 (24.4-32.4)
cPRA	Zero	91326 (57.0)	35757 (62)	40188 (55)	15381 (54)
	Positive	68999 (43.0)	22119 (38)	33524 (45)	13356 (46)
Positive cPRA level	%	57.8 (21.8-91.8)	58.5 (23.2-87.6)	58.0 (21.4-95.3)	57.5 (19.2-92.2)
Primary Diagnosis	Type I diabetes	4048 (2.5)	1925 (3)	1530 (2)	593 (2)
	Type II diabetes	41210 (25.7)	13746 (24)	19262 (26)	8202 (29)
	HTN	38460 (24.0)	14132 (24)	17515 (24)	6813 (24)
	PKD	11848 (7.4)	4683 (8)	5270 (7)	1895 (7)
	Graft failure	10886 (6.8)	3794 (7)	5345 (7)	1747 (6)
	IgA nephropathy	6553 (4.1)	2011 (3)	3217 (4)	1325 (5)
	SLE	4643 (2.9)	1575 (3)	2233 (3)	835 (3)
	Other	42677 (26.6)	16010 (28)	19340 (26)	7327 (25)
Previous Kidney Tx	Yes	19013 (11.9)	7069 (12)	8961 (12)	2983 (10)
Diabetes history	Yes	58545 (36.5)	20936 (36)	26674 (36)	10935 (38)
Dialysis	Pre-emptive	16261 (10.1)	5967 (10)	6952 (9)	3342 (12)
	HD/PD	144064 (89.9)	51909 (90)	66760 (91)	25395 (88)
HD/PD Dialysis	Days	1487 (817-2295)			
Functional Status	10 to 40%	3625 (2.3)	1001 (2)	1938 (3)	686 (2)
	50 to 70%	65715 (41.0)	17667 (31)	34892 (47)	13156 (46)
	80 to 100%	90985 (56.8)	39208 (68)	36882 (50)	14895 (52)
HLA mismatch	0	10268 (6.4)	5125 (9)	3730 (5)	1413 (5)
	1 to 4	75467 (47.1)	25854 (45)	35681 (48)	13932 (48)
	5 to 6	74590 (46.5)	26897 (46)	34301 (47)	13392 (47)

Induction Group	Depleting	115081 (71.8)	36819 (64)	55641 (75)	22621 (79)
	Nondepleting	24631 (15.4)	10608 (18)	10247 (14)	3776 (13)
	Both	4207 (2.6)	1758 (3)	1940 (3)	509 (2)
	None	16406 (10.2)	8691 (15)	5884 (8)	1831 (6)
Transplant Year	Dec 5-31, 2007	549 (0.3)	549 (1)		
	2008	7413 (4.6)	7413 (13)		
	2009	7542 (4.7)	7542 (13)		
	2010	7919 (4.9)	7919 (14)		
	2011	8538 (5.3)	8538 (15)		
	2012	8441 (5.3)	8441 (15)		
	2013	8875 (5.5)	8875 (15)		
	Jan 1-Dec 3, 2014<br>Dec 4-Dec 31, 2014	9252 (5.8)	8599 (15)	653 (1)	
	2015	9608 (6.0)		9608 (13)	
	2016	10509 (6.6)		10509 (14)	
	2017	11169 (7.0)		11169 (15)	
	2018	11677 (7.3)		11677 (16)	
	2019	13235 (8.3)		13235 (18)	
	2020	14076 (8.8)		14076 (19)	
	2021	14852 (9.3)		2785 (4)	12067 (42)
	2022	15623 (9.7)			15623 (54)
	Jan 1-25, 2023	1047 (0.7)			1047 (4)
**205 Centers Across Up To 17 Years**					
Annualized Center Volume		48 (26-88)	41 (24-70)	65 (31-138)	73 (40-145)

Abbreviations: KAS, kidney allocation system; KAS250, kidney allocation system 250 NM; HLA mm, human leukocyte antigen mismatch; cPRA, calculated panel reactive antibody; HTN, hypertension; PKD, polycystic kidney disease; HD, hemodialysis; PD, peritoneal dialysis; IgA, immunoglobulin A; SLE, systemic lupus erythematous.

Table S6. Multivariate Cox Regression Results Based on Standardized Data

Variable	Std Dev	HR and 95% CI	p-value	Rank
Recipient Age, years	13	1.228 (1.215,1.241)	<0.0001	1
Donor Age, years	16	1.175 (1.162,1.188)	<0.0001	2
Recipient Ethnicity (ref=White)			<0.0001	3
Black		0.994 (0.974,1.015)	0.5985	
Hispanic/Latino		0.743 (0.723,0.763)	<0.0001	
Other		0.712 (0.688,0.737)	<0.0001	
Days on Dialysis	1262	1.120 (1.111,1.130)	<0.0001	4
Diagnosis at Time of Listing (ref= Type II diabetes)			<0.0001	5
Type I diabetes		1.029 (0.981,1.081)	0.2407	
Hypertension		0.943 (0.913,0.974)	0.0004	
Polycystic Kidney Disease		0.644 (0.615,0.675)	<0.0001	
Graft failure		0.879 (0.832,0.928)	<0.0001	
IgA nephropathy		0.654 (0.614,0.696)	<0.0001	
Systemic Lupus Erythematous		1.038 (0.975,1.105)	0.2397	
Other		0.913 (0.883,0.944)	<0.0001	
Transplant Year	4.4	0.891 (0.881,0.901)	<0.0001	6
Recipient Diabetes (ref=No)		1.308 (1.270,1.346)	<0.0001	7
Functional Status (ref=80% to 100%)			<0.0001	8
10% to 40%		1.272 (1.206,1.342)	<0.0001	
50% to 70%		1.126 (1.106,1.146)	<0.0001	
Donor Diabetes (ref=No)		1.227 (1.192,1.263)	<0.0001	9
Recipient Gender (ref=Female)		0.886 (0.870,0.903)	<0.0001	10
Donor Ethnicity (ref=White)			<0.0001	11
Black		1.143 (1.115,1.171)	<0.0001	
Hispanic/Latino		0.946 (0.922,0.972)	<0.0001	
Other		0.981 (0.936,1.029)	0.4308	
Recipient Body Mass Index, kg/m^2	5.4	1.055 (1.046,1.064)	<0.0001	12
Donor Hypertension History (ref=No)		1.122 (1.099,1.145)	<0.0001	13
Induction Group (ref=Depleting)			<0.0001	14
Non-Depleting		1.032 (1.008,1.056)	0.0083	
Depleting & Non-Depleting		1.129 (1.076,1.184)	<0.0001	
None		1.144 (1.115,1.173)	<0.0001	
Human leukocyte antigen mismatch (ref=0)			<0.0001	15
1 to 4		1.147 (1.104,1.192)	<0.0001	
5 to 6		1.221 (1.173,1.270)	<0.0001	
Donor Height, cm	17.9	0.942 (0.930,0.954)	<0.0001	16
Calculated panel reactive antibody, %	36.2	1.048 (1.037,1.060)	<0.0001	17
Non-Heart Beating Donor (ref=No)		1.097 (1.072,1.122)	<0.0001	18
Allocation Type (ref=Local)			<0.0001	19
Regional		1.061 (1.033,1.090)	<0.0001	
National		1.090 (1.058,1.122)	<0.0001	
Donor cause of death (ref=Anoxia)			<0.0001	20
Cerebrovascular /Stroke		1.051 (1.028,1.075)	<0.0001	
Head Trauma		0.984 (0.962,1.006)	0.1433	
CNS Tumor		0.918 (0.806,1.046)	0.1969	
Other		1.065 (1.012,1.121)	0.0151	

Variable	Std Dev	HR and 95% CI	p-value	Rank
Previous Kidney Transplant (ref=No)		1.102 (1.059,1.146)	<0.0001	21
Cold Ischemia Time Range (ref=4 to <8), hours			<0.0001	22
8 to <12		0.964 (0.931,0.999)	0.0418	
12 to <16		1.008 (0.974,1.042)	0.6620	
16 to <20		0.997 (0.963,1.032)	0.8643	
20 to <24		1.032 (0.995,1.071)	0.0866	
24 to <28		1.031 (0.990,1.074)	0.1354	
28 to <32		1.025 (0.977,1.077)	0.3120	
32 to <36		1.097 (1.037,1.162)	0.0014	
Annualized Center Volume, n	68.7	0.979 (0.969,0.989)	<0.0001	23
Donor Creatinine, mg/dl	1.1	1.018 (1.008,1.028)	0.0002	24
Donor Weight, kg	25.3	0.981 (0.970,0.992)	0.0007	25
Expedited Placement (ref=No)		0.952 (0.890,1.017)	0.1458	26
Donor Hepatitis C Virus Antibody (ref=Negative)		1.030 (0.988,1.074)	0.1623	27
Donor Gender (ref=Male)		0.994 (0.975,1.013)	0.5451	28
Kidney Pumped (ref=No)		1.006 (0.987,1.025)	0.5561	29

Table S7 Baseline recipient characteristics comparing non-expedited and expedited placement kidneys in the KAS250 era, and expedited placement kidneys comparing the KAS and KAS 250 eras.

		KAS 250 (N=28,737)			Expedited Placement Kidneys (N=4,767)		
**Variable**	**Parameter Value**	Not Expedited (n=25,039, 87.1%)	Expedited (n=3,698, 12.9%)	P-value	KAS era (n=1,069, 1.5%)	KAS250 era (n=3,698, 12.9%)	P-Value
**Recipient demographics**							
Recipient age	Years	55 (43-64)	59 (49-67)	<.0001	61 (51-68)	59 (49-67)	0.0004
Recipient gender	Female	10112 (40)	1375 (37)	0.0002	393 (37)	1375 (37)	0.8028
	Male	14927 (60)	2323 (63)		676 (63)	2323 (63)	
Recipient ethnicity	White	8394 (34)	1378 (37)	<.0001	403 (38)	1378 (37)	<.0001
	Black	8865 (35)	1219 (33)		242 (23)	1219 (33)	
	Hispanic/Latino	5278 (21)	667 (18)		266 (25)	667 (18)	
	Other	2502 (10)	434 (12)		158 (15)	434 (12)	
BMI	Kg/m^2	28.2 (24.5-32.6)	27.8 (24.3-31.7)	<.0001	27.0 (24.1-30.7)	27.8 (24.3-31.7)	0.0006
cPRA	Negative	12983 (52)	2398 (65)	<.0001	741 (69)	2398 (65)	0.0066
	Positive	12056 (48)	1300 (35)		328 (31)	1300 (35)	
Positive cPRA level	%	59.9 (21.9-93.0)	36.5 (6.9-77.3)	<.0001	29.2 (6.1-73.5)	36.5 (6.9-77.3)	0.3789
Primary diagnosis	Type I diabetes	529 (2)	64 (2)	<.0001	21 (2)	64 (2)	0.5371
	Type II diabetes	6944 (28)	1258 (34)		362 (34)	1258 (34)	
	HTN	5956 (24)	857 (23)		219 (20)	857 (23)	
	PKD	1625 (6)	270 (7)		81 (8)	270 (7)	
	Graft failure	1613 (6)	134 (4)		33 (3)	134 (4)	
	IgA nephropathy	1150 (5)	175 (5)		58 (5)	175 (5)	
	SLE	760 (3)	75 (2)		24 (2)	75 (2)	
	Other	6462 (26)	865 (23)		271 (25)	865 (23)	
Previous kidney transplant	Yes	2757 (11)	226 (6)	<.0001	64 (6)	226 (6)	0.8808
Diabetes history	Yes	9348 (37)	1587 (43)	<.0001	462 (43)	1587 (43)	0.8602
Dialysis	Pre-emptive	2780 (11)	562 (15)	<.0001	147 (14)	562 (15)	0.2418
	HD/PD	22259 (89)	3136 (85)		922 (86)	3136 (85)	
HD/PD Dialysis Time	Days	1577 (834-2362)	1051 (535-1759)	<.0001	1031 (537-1723)	1051 (535-1759)	0.8098

Functional Status	10 to 40%	628 (3)	58 (2)	<.0001	13 (1)	58 (2)	0.3912
	50 to 70%	11563 (46)	1593 (43)		482 (45)	1593 (43)	
	80 to 100%	12848 (51)	2047 (55)		574 (54)	2047 (55)	
HLA mismatch	0	1322 (5)	91 (2)	<.0001	31 (3)	91 (2)	0.5123
	1 to 4	12319 (49)	1613 (44)		449 (42)	1613 (44)	
	5 to 6	11398 (46)	1994 (54)		589 (55)	1994 (54)	
Induction Group	Depleting	19660 (79)	2961 (80)	<.0001	663 (62)	2961 (80)	<.0001
	Nondepleting	3330 (13)	446 (12)		203 (19)	446 (12)	
	Both	398 (2)	111 (3)		149 (14)	111 (3)	
	None	1651 (7)	180 (5)		54 (5)	180 (5)	
Total time on wait list	days	525 (110-1328)	374 (92-1016)	<.0001	428 (125-1078)	374 (92-1016)	0.0151

Abbreviations: KAS, kidney allocation system; KAS250, kidney allocation system 250 NM; BMI, body mass index; HLA mm, human leukocyte antigen mismatch; cPRA, calculated panel reactive antibody; HTN, hypertension; PKD, polycystic kidney disease; HD, hemodialysis; PD, peritoneal dialysis; IgA, immunoglobulin A; SLE, systemic lupus erythematous
